# Using Citizen Science to Scout Honey Bee Colonies That Naturally Survive *Varroa destructor* Infestations

**DOI:** 10.3390/insects12060536

**Published:** 2021-06-09

**Authors:** Arrigo Moro, Alexis Beaurepaire, Raffaele Dall’Olio, Steve Rogenstein, Tjeerd Blacquière, Bjørn Dahle, Joachim R. de Miranda, Vincent Dietemann, Barbara Locke, Rosa María Licón Luna, Yves Le Conte, Peter Neumann

**Affiliations:** 1 Institute of Bee Health, Vetsuisse Faculty, University of Bern, 3003 Bern, Switzerland; alexis.beaurepaire@vetsuisse.unibe.ch (A.B.); peter.neumann@vetsuisse.unibe.ch (P.N.); 2 BeeSources, 40132 Bologna, Italy; raffaele.dallolio@gmail.com; 3 The Ambeessadors, 10439 Berlin, Germany; ambeessadors@gmail.com; 4 Wageningen Plant Research, Wageningen University & Research, 6708 PB Wageningen & Blacqbee, The Netherlands; blacqbee@blacqbee.nl; 5 Norwegian Beekeepers Association, NO-2040 Kløfta, Norway; bjorn.dahle@norbi.no; 6 Faculty of Environmental Sciences and Natural Resource Management, Norwegian University of Life Sciences, 1433 Ås, Norway; 7 Department of Ecology, Swedish University of Agricultural Sciences, 750 07 Uppsala, Sweden; joachim.de.miranda@slu.se (J.R.d.M.); barbara.locke@slu.se (B.L.); 8 Swiss Bee Research Center, Agroscope, 3003 Bern, Switzerland; vincent.dietemann@agroscope.admin.ch; 9 Department of Ecology and Evolution, Biophore, UNIL-Sorge, University of Lausanne, 1015 Lausanne, Switzerland; 10 Wild Bees Project, 01170 Crozet, France; romaliconluna@live.com; 11 UR 406 Abeilles et Environnement, INRAE, 84914 Avignon, France; yves.le-conte@inrae.fr

**Keywords:** Citizen Science, COLOSS, honey bee, Honey Bee Watch, monitoring, natural selection, *Varroa destructor*

## Abstract

**Simple Summary:**

Citizen Science is a valuable resource that can substantially contribute to the conservation of biodiversity. However, its use in honey bee research has remained minimal. The Survivors Task Force of the COLOSS association created and promoted an online surveying tool with the aim of identifying potential cases of Western honey bee, *Apis mellifera*, populations that are surviving infestations with ectoparasitic mites *Varroa destructor* without control measures by beekeepers. The reports suggest that there could be twice as many naturally surviving colonies worldwide than are currently known. The survey also shows that citizens can be readily engaged through social media, personal networks, and promotional campaigns to gather valuable and previously inaccessible data. These reports of surviving honey bee colonies will now be validated through the new initiative Honey Bee Watch, a global and multi-year Citizen Science project founded to connect citizens, beekeepers, and scientists. This will enable to increase scientific knowledge, mitigate honey bee colony losses, and develop education and conservation campaigns.

**Abstract:**

Citizen Science contributes significantly to the conservation of biodiversity, but its application to honey bee research has remained minimal. Even though certain European honey bee (*Apis* *mellifera*) populations are known to naturally survive *Varroa destructor* infestations, it is unclear how widespread or common such populations are. Such colonies are highly valuable for investigating the mechanisms enabling colony survival, as well as for tracking the conservation status of free-living honey bees. Here, we use targeted Citizen Science to identify potentially new cases of managed or free-living *A. mellifera* populations that survive *V. destructor* without mite control strategies. In 2018, a survey containing 20 questions was developed, translated into 13 languages, and promoted at beekeeping conferences and online. After three years, 305 reports were collected from 28 countries: 241 from managed colonies and 64 from free-living colonies. The collected data suggest that there could be twice as many naturally surviving colonies worldwide than are currently known. Further, online and personal promotion seem to be key for successful recruitment of participants. Although the survivor status of these colonies still needs to be confirmed, the volume of reports and responses already illustrate how effectively Citizen Science can contribute to bee research by massively increasing generated data, broadening opportunities for comparative research, and fostering collaboration between scientists, beekeepers, and citizens. The success of this survey spurred the development of a more advanced Citizen Science platform, Honey Bee Watch, that will enable a more accurate reporting, confirmation, and monitoring of surviving colonies, and strengthen the ties between science, stakeholders, and citizens to foster the protection of both free-living and managed honey bees.

## 1. Introduction

Citizen Science is an effective tool for engaging the general public in research projects. It is most commonly used in media-friendly subjects such as ecology and conservation [1,2,3,4]. By definition, this discipline relies on the active involvement of the public in the provision of data and the co-creation of scientific knowledge [5,6]. For scientists, this offers many opportunities, such as real-time access to large-scale data and direct contact with both citizens and practitioners [7]. Citizen Science simultaneously offers citizens the opportunity to partake in research questions that interest them, while also providing the possibility of advancing their education [8], with multiple benefits to all actors involved [9,10]. As such, the use of Citizen Science for facing the multiple challenges affecting global biodiversity has been widely increasing, even in bee research [11]. However, despite the need to mitigate the current global health crisis affecting Western honey bees, *A. mellifera* [12,13], Citizen Science almost exclusively focused on the quantification of winter losses of managed colonies [11] and has not yet been capitalized for finding possible solutions to this problem.

Severe losses of managed colonies of Western honey bees have in fact been thoroughly monitored in the last decades [14] and the ectoparasitic mite *Varroa destructor* has been widely recognized as one of the major drivers of these losses. Generally, infestations with this parasite lead to the death of a colony within two years unless appropriate control measures are taken [15]. Beekeepers worldwide therefore rely on mite control measures (primarily regular acaricide treatments), in order to keep their stocks alive [16]. However, these acaricides vary in efficacy, are prone to resistance development by the mites and contaminate hive products and can thus only be used outside the foraging season [17]. As it stands, chemical treatments therefore do not represent a long-term solution to *V. destructor* [17]. Non-chemical alternative treatments have also been developed [18], but are currently not widely used and represent an increased work load for the beekeepers. The discovery of naturally *V. destructor*-surviving populations [19,20] led to the realization that the Western honey bee possesses certain traits enabling their survival without the need for mite control [21], similar to what is observed in the original host *Apis cerana* [22]. This encouraged scientists and beekeepers to search for or establish *A. mellifera* populations capable of surviving *V. destructor* infestation without mite control [20] in order to identify traits that are most amenable to natural or artificial selection [19,21,23]. Unfortunately, identifying or establishing such populations takes much time and effort, leading to a research that focuses on only a few populations [19]. However, considering a more diverse group of *V. destructor*-surviving *A. mellifera* populations that undoubtedly exist [24] would provide increased opportunities to investigate known survival mechanisms and discover novel ones. Moreover, a diversity of naturally surviving populations could represent an important asset for the re-establishment of *A. mellifera* in the wild. 

European *A. mellifera* populations have been considered almost extinct in the wild as a consequence of the spread of *V. destructor* [25]. However, recent evidence suggests that free-living honey bee colonies can survive in the wild in a self-sustainable manner [26,27,28,29,30]. Despite these few occurrences, there remains a large gap of knowledge on the current abundance, distribution, and diversity of free-living *A. mellifera* populations. As their identification is most efficiently achieved with large-scale coordinated efforts, it appears high time to mobilize Citizen Scientists for a large-scale survey on this topic.

Beekeepers represent the major stakeholders upon which honey bee health ultimately depends [31,32]. Their participation in bee health research is therefore both desirable and mutually beneficial. Furthermore, the recent development of online surveying platforms makes it much easier to involve stakeholders such as beekeepers as well as the general public in participatory research [33]. As the level of contribution among users of such platforms is often uneven [7], the use of multiple communication channels, including social media and newsletters, can foster wider and more representative engagement by citizens [34]. Here, we present the outcome of an online survey organized by the members of “Survivors”, a Task Force within the COLOSS (Prevention of honey bee COlony LOSSes; www.coloss.org, accessed on 7 June 2021) association, aimed at both beekeepers and the general public, for mapping and identifying new cases of *A. mellifera* colonies that, either in the wild or in managed apiaries, survive *V. destructor* infestation without the need for mite control.

## 2. Materials and Methods

**Figure 1:**
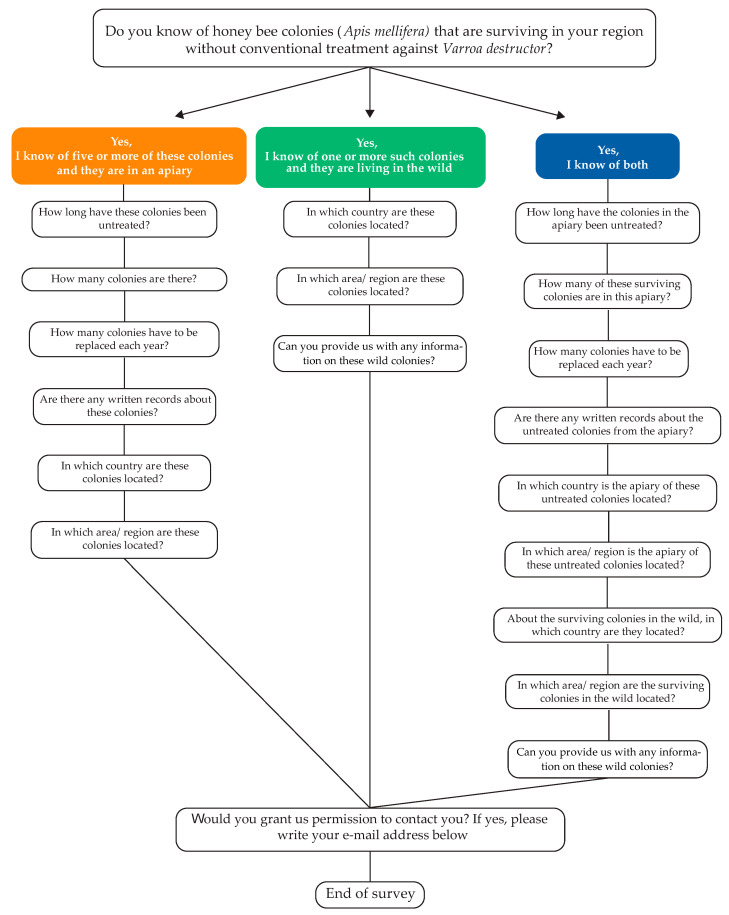
Flow diagram of the online survey on honey bee, *Apis mellifera*, colonies surviving infestations with *Varroa destructor* without acaricidal treatments. The first question directed respondents to one of three sections, depending on whether they intended to report cases of managed *A. mellifera* colonies surviving without conventional treatment against *V. destructor* (left), free-living surviving *A. mellifera* colonies (middle), or both (right).

In March 2018, during a COLOSS Survivors Task Force workshop in Bern, Switzerland, an online survey was created (Figure 1) and later translated into 13 languages: Chinese, Dutch, English, French, German, Italian, Norwegian, Russian, Serbian, Slovenian, Spanish, Swedish, and Ukrainian. The survey was developed using the Google^®^ Form platform, activated and made accessible from the Survivors Task Force webpage (https://coloss.org/taskforces/survivors/, accessed on 7 June 2021). The survey was simultaneously disseminated through local national beekeeping networks of the COLOSS Survivors Task Force members through a variety of channels. The initiative was further promoted during a beekeeping conference in the Netherlands in 2018, followed by a passive online campaign using social networks.

The survey started with an introductory question asking the responder for possible cases of surviving *A. mellifera* populations (Figure 1). As the survey aimed at identifying surviving honey bee populations, rather than individual colonies, a stipulation was included to only submit reports of a minimum of five surviving colonies from managed beekeeping operations. This requirement was not imposed for free-living survivors, which inevitably are well-dispersed individual colonies, wherever they are found (Figure 1). Depending on the answer to the introductory question, the user was directed to one of three sections (Figure 1). The first section concerned managed surviving colonies and contained seven questions. These questions aimed at collecting data regarding the general location of each case (i.e., region and city name), the number of years these colonies was known to survive without mite control, the number of colonies in the surviving group at the date of the report, and finally the proportion of colonies that needed to be replaced annually to maintain the population size, as a measure of the population’s ability to survive *V. destructor* unaided. The second section concerned possible cases of free-living survivors and contained a single question about the general location of the colony, together with an open-text field to provide other relevant information. The third section combined questions from Section 1 and Section 2, for users reporting cases of both managed and free-living surviving colonies. At the end of the survey, the user was given the possibility to submit a personal contact, for future case validation and collaborative research (Figure 1). The respondent’s personal data were treated confidentially, in compliance with the General Data Protection Regulations (GDPR) of the European Union [35].

In January 2021, the reports were compiled and screened to remove duplicate reports and cases already known to science. The compiled data set was analyzed statistically with respect to a range of factors relevant to the aims of the project, using R software [36]. For potentially stable surviving populations of managed survivors, the reports were ranked in three classes: “gold”, “silver”, and “bronze”, according to the reported survival time, the number of colonies in the group, and the annual proportion of colony replacement. For all classes, the minimum requirement for inclusion was an annual replacement rate of less than 50%. Cases reporting more than 30 colonies surviving for more than 10 years were considered as the “gold” standard. Next, groups surviving more than 10 years but involving fewer than 30 colonies, were considered silver class. Last, groups of more than 30 colonies surviving for a period between five and ten years, were considered as bronze class. All reports falling outside these criteria were included into a fourth class.

Additional voluntary information added to reports on free-living colonies were analyzed qualitatively.

## 3. Results

**Figure 2:**
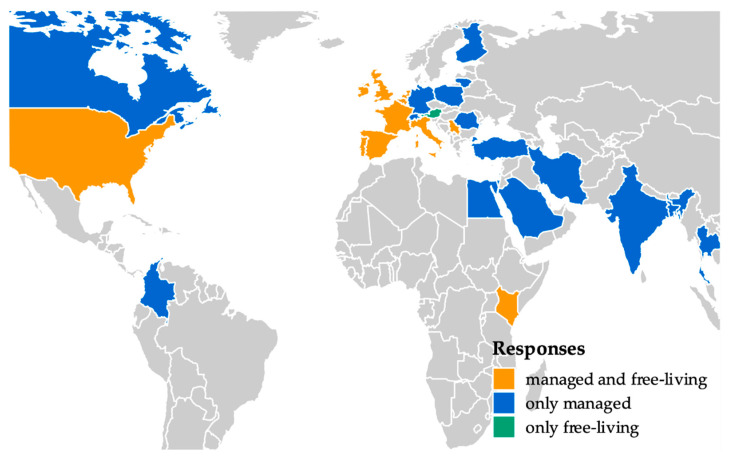
Global reach of the COLOSS Survivors Task Force online survey on possible cases of honey bee, *Apis mellifera*, colonies that, in an apiary or free-living, are surviving *Varroa destructor* mite infestations in the absence of chemical treatments. Regions indicated by respondents are highlighted with different colors, depending on the type of surviving colonies (managed, only free-living, or both). The majority of reports were submitted from UK, The Netherlands, and USA.

In total, 305 reports were collected from 28 countries (Figure 2; Table 1), comprising 64 reports on free-living colonies and 241 on managed colonies. The majority of users provided a contact for future case confirmation (N = 216, 86%). Most of the reports were from the United Kingdom (N = 86, 28%), The Netherlands (N = 77, 25%), and the USA (N = 68, 22%).

Overall, only a few reports were submitted on possible cases of free-living colonies (N = 64, 20%, Table 1). Respondents consistently provided the general locations in which these colonies were located (Table 1), and a large proportion also provided voluntary additional information on each case (N = 48, 75%). From this information, a count of the reports that mentioned the type of nests in which the free-living colonies resided (N = 35, 72%) or whether the respondent monitored these nests (N = 20, 41%) could be extracted purposefully (Table 2).

**Figure 3:**
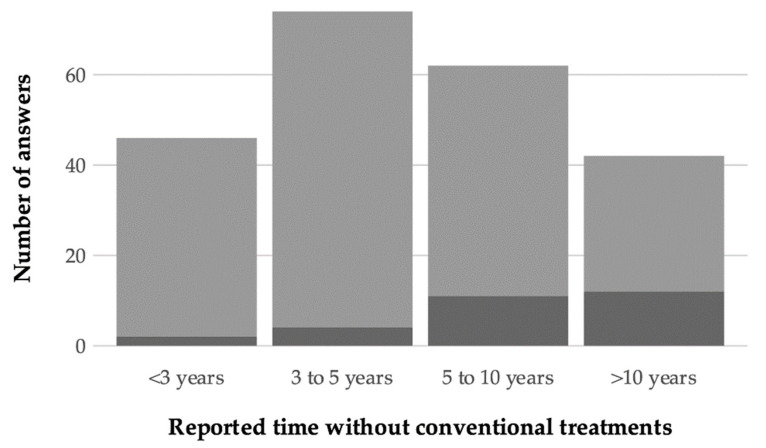
Time span over which the reported managed honey bee, *Apis mellifera*, colonies were not subjected to treatments against *Varroa destructor*. The number of answers from the COLOSS survey is shown (dark gray = surviving populations with >30 colonies, N = 29; light gray = groups between five and 30 colonies, N = 195).

When reporting about managed surviving colonies (Total N = 241), almost all respondents (N = 224, 93%) indicated the number of colonies composing the group. Among these, 195 (87%) described groups of five to 30 colonies, 44 (22%) of which untreated and surviving for less than three years, 70 (36%) for a period between three and five years, 51 (26%) for a period between five and 10 years, and 30 (15%) for more than 10 years (Figure 3). Few respondents described managed groups consisting of more than 30 colonies (N = 29), of which 6 (20%) were untreated and surviving for less than five years, 11 (37%) for a period between five and 10 years, and 12 (41%) for more than 10 years (Figure 3).

**Figure 4:**
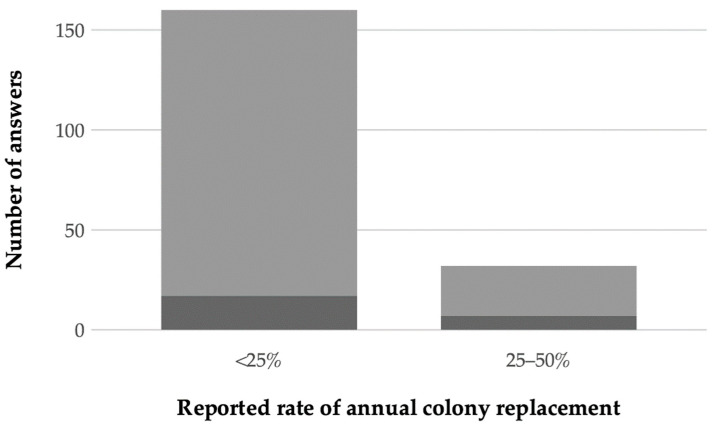
Annual colony replacement rate in groups of managed honey bee, *Apis mellifera*, colonies not treated against parasitic mites *Varroa destructor*. The number of answers from the COLOSS Survivors Task Force survey is shown (dark gray = groups with >30 colonies, N = 26; light gray = groups between five and 30 colonies, N = 174).

Respondents reporting of managed surviving colonies also indicated the proportion of colonies that needed to be replaced annually in order to maintain the stock (Figure 4). Among these, the majority (N = 160, 80%) reported replacing less than one-quarter of the colonies per year (Figure 4), while the remaining respondents reported replacing between one-quarter and one-half of the colonies in their stock (N = 32, 16%, Figure 4) per year. Another eight (4%) reports indicated an annual replacement rate of more than one-half. Because these high rates suggest that these colonies did not develop a stable relationship with *V. destructor*, these eight reports were not considered as potential survivors and were excluded.

**Figure 5:**
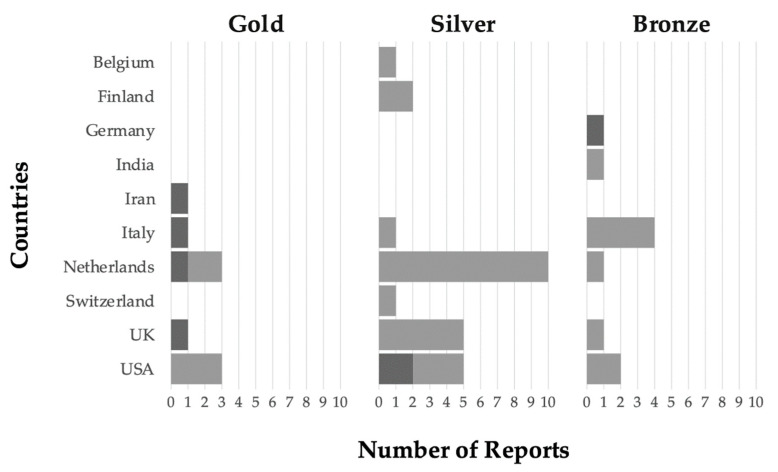
Counts of reports on potentially self-sustaining managed *A. mellifera* populations surviving *Varroa destructor* based on group composition, proportion of annual colony replacement, and untreated period as collected by the COLOSS Survivors Task Force survey. The reports are divided by classes (gold, silver, and bronze) and regions. Dark gray = cases for which a replacement rate between 25 and 50% was reported; light gray = cases for which a replacement rate lower than 25% was reported. Gold class (N = 9) was defined by managed groups of colonies composed of more than 30 colonies with an annual replacement rate below 50% and untreated for more than 10 years. Silver class (N = 25) were managed groups of colonies composed of a number between five and 30 colonies with an annual replacement rate below 50% and untreated for more than 10 years. Bronze class (N = 10) were managed groups of colonies composed of more than 30 colonies with an annual replacement rate below 50% and untreated for a period between five and 10 years. Only cases previously unknown to scientific literature have been included.

Interestingly, 44 reports (18%) collected on managed, untreated groups could be classified as potentially stable populations of survivors as nine gold, 25 silver, and 10 bronze cases were found (Figure 5).

## 4. Discussion

By providing access to previously unreported cases of untreated *A. mellifera* colonies potentially surviving *V. destructor* infestations without treatments, this survey will help improve our understanding of the mechanisms underlying survival of colonies. The output of this survey further illustrates the potential of Citizen Science to provide valuable and large-scale data for solving the major health problems that Western honey bees are currently facing worldwide. 

Despite a low investment in online and personal communications to promote the survey, its outreach was substantial. Notably, it engaged responders from continents not included in previous COLOSS surveys [14]. Interestingly, the majority of reports were collected from three countries: United Kingdom, the Netherlands, and the USA (Table 1). This pattern seems to largely stem from the way by which the survey was promoted. Most answers were submitted after the authors personally promoted the survey during a conference held in the Netherlands in 2018, at which attendees were mostly local or from the UK and USA. Following this event, a modest social media campaign was launched to further promote the survey within the conference attendee’s networks. Moreover, in the same period, the link to the survey was also spread to Dutch beekeepers through an online newsletter. As has been the case for other citizen science initiatives [6], the recruitment of participants through personal engagement and the use of online and social media platforms appeared to have been crucial for the successful dissemination of this initiative.

The survey of the COLOSS Survivors Task Force lists among the few scientific initiatives aimed at mapping free-living and surviving *A. mellifera* colonies on an international scale [30]. In the northern hemisphere, free-living colonies are considered to be very rare [25,37] and are notoriously difficult to spot in the field [28]. As a consequence, few reports (i.e., 20% of the total answers, Table 1) were collected on such cases in comparison to managed colonies. The data collected on free-living colonies provided only an indication of their locations (Table 1). A comparative analysis of the data derived from these cases was not possible given that the majority of information collected consisted of anecdotal reports. Most likely, this was due to the lack of precise instructions given to users when submitting information on such colonies (Figure 1). Yet, more than one-third of the responses collected suggested that responders were voluntarily monitoring the free-living colonies known to them (Table 2). This considerable level of public engagement is promising and suggests that there are good perspectives for successfully implementing a more advanced version of this Citizen Science tool capable of obtaining more detailed data on free-living honey bee colonies. This is the goal of a follow-up initiative developed by a core team of members within the COLOSS Survivors Task Force. The team launched Honey Bee Watch (www.honeybeewatch.com, accessed on 7 June 2021), which aims at pursuing this study in greater depth, over a much longer timeframe, and with a much wider scope that includes all *Apis* species so to provide more data over their distribution and conservation status [38,39].

The potential cases of survivors managed by beekeepers collected in the survey may substantially contribute to the number of previously known cases of untreated populations of Western honey bees surviving *V. destructor* infestation. Current scientific literature indicates that approximately 20 untreated and surviving populations are managed by beekeepers/breeders or are part of scientific projects [20,40,41,42,43,44,45,46,47,48,49,50,51]. The answers collected from this survey reported twice as many cases, the majority of which in regions with no previously reported cases (Figure 5). The data also suggest that some of these honey bee colonies may have reached a stable equilibrium with *V. destructor*, as the majority of respondents reported an annual colony replacement rate <25% (Figure 5). This indicates adaptations of both the honey bee host to the selection pressure imposed by the parasite [23,43] and the local mites to its host [52]. Yet, despite the promising data obtained, these potential novel cases need to be confirmed via thorough investigation and long-term monitoring before they can be considered as surviving mite infestations. With each respondent’s approval, and after funding has been secured, phenotypic and molecular tests will be performed by the Honey Bee Watch study on gold, silver, and bronze cases. Inspired by the level of citizens’ engagement that the present initiative generated, Honey Bee Watch will initiate a more strategically focused communication campaign to continue collecting data on untreated and free-living honey bee colonies.

## 5. Conclusions

Given the relevant contributions that Citizen Science initiatives have demonstrated in multiple conservation and ecological studies [2], using this tool to investigate the extent of *A. mellifera* populations surviving *V. destructor* without treatments appears both meaningful and fruitful. Through the COLOSS Survivors Task Force survey, beekeepers, and citizens, incentivized by social media and promotional campaigns, were motivated to submit data on and monitor untreated and free-living colonies. In the process, they have become a valuable reporting resource on potentially self-sustainable and *V. destructor* surviving *A. mellifera* populations. As the initiative reported here has ended, the results gathered are calling for case validation and the development of a more advanced citizen science platform. To fulfil such aims, COLOSS Survivors Task Force members initiated Honey Bee Watch (www.honeybeewatch.com, accessed on 7 June 2021), aimed at expanding data collection on untreated, surviving, and free-living honey bee colonies. Overall, this initiative, together with the results obtained from the scientific validation of the cases presented here, may ultimately demonstrate how bridging the gap between scientists, practitioners, and citizens can help discover solutions to promote large-scale conservation of biodiversity.

## Figures and Tables

**Table 1:** Number of reports, divided per category, collected by the COLOSS Survivors Task Force survey on putative cases of untreated *A. mellifera* colonies surviving *V. destructor* infestation. The country in which these colonies were reported to occur is specified.

Category of Answer	Number of Answers	Countries
Managed survivors	241	Bangladesh (1), Belgium (12), Canada (3), Colombia (1), Egypt (2), Finland (2), France (1), Germany (1), India (3), Iran (1), Ireland (1), Israel (1), Italy (16), Kenya (1), Lithuania (3), Netherlands (62), Poland (1), Portugal (4), Romania (1), Saudi Arabia (1), Serbia (1), Spain (1), Switzerland (3), Thailand (1), Turkey (1), UK (63), USA (53)
Free-living survivors	64	Austria (1), Belgium (1), France (1), Ireland (2), Italy (1), Kenya (1), Netherlands (15), Portugal (2), Serbia (1), Spain (1), UK (23), USA (15)
Total	305	

**Table 2:** Counts and proportions of users who did or did not submit case-specific information on free-living colonies to the COLOSS Survivors Task Force survey. In total, 64 reports on free-living colonies were submitted. The counts and proportions of instances in which the type of nests was mentioned are also given, together with those in which the user reported to be voluntarily monitoring the colonies.

Types of Response	Number of Answers	Percentage
Reported information	48	75%
No reported information	16	25%
Described nest type	35	54%
Voluntary monitoring	20	31%

## Data Availability

Data used in the submitted manuscript can be available after reasonable request to the corresponding author. Personal data of survey respondents will not be provided as it would violate privacy laws.
